# MiRNA-144-3p inhibits high glucose induced cell proliferation through suppressing FGF16

**DOI:** 10.1042/BSR20181788

**Published:** 2019-07-26

**Authors:** Cuimin Chen, Chunyan Zhao, Cao Gu, Xiao Cui, Jinhui Wu

**Affiliations:** Departments of Ophthalmology, Changhai Hospital, Second Military Medical University, Shanghai 200433, China

**Keywords:** cell proliferation, diabetic retinopathy, FGF16, miR-144-3p

## Abstract

As a major cause of blindness, diabetic retinopathy (DR) is often found in the developed countries**.** Our previous study identified a down-regulated miRNA: miR-144-3p in response to hyperglycemia. The present study aims to investigate the role of miR-144-3p in proliferation of microvascular epithelial cells. Endothelial cells were treated with different concentrations of glucose, after which miR-144-3p were detected with real-time PCR assay. MiR-144-3p mimics or inhibitors were used to increase or knockdown the level of this miRNA. Western blotting assay and ELISA assay were used to measure the expression and concentration of VEGF protein. 5-Bromo-2-deoxyUridine (BrdU) labeled cell cycle assay was used to detect cells in S phase. MiRNA targets were predicted by using a TargetScan tool, and were further verified by luciferase reporter assay. In the present study, we focussed on a significantly down-regulated miRNA, miR-144-3p, and investigated its role in high glucose (HG) induced cell proliferation. Our data showed that miR-144-3p mimics significantly inhibited HG induced cell proliferation and reduced the percentage of cells in S phase. HG induced up-regulation of VEGF was also prohibited by miR-144-3p mimics. Through wound-healing assay, we found that miR-144-3p suppressed cell migration after HG treatments. Moreover, we predicted and proved that fibroblast growth factor (FGF)16 is a direct target of miR-144-3p. Finally, miR-144-3p attenuated HG induced MAPK activation. In conclusion, we demonstrated that miR-144-3p inhibited high glucose-induced cell proliferation through suppressing FGF16 and MAPK signaling pathway, suggesting a possible role of miR-144-FGF16 in the development of DR.

## Introduction

As one of the most common complications of diabetes, diabetic retinopathy (DR) is a major cause of blindness, especially in the developed countries [1,2]. Hyperglycemia is the initial pathogenic factor of retinal pathological changes, including DR and the breakdown of blood-retinal barrier [3]. Amongst all these features, abnormal proliferation of retinal microvascular endothelial cell (REC) resulted from hyperglycemia is considered as a hallmark of proliferative DR (PDR) [4]. However, even the roles of VEGF and other factors have been identified, the underlying mechanism of vascular endothelial cell proliferation in DR remains unclear.

MiRNAs are a class of single strand noncoding RNA (20–25 nts), regulating biological processes through a post-translational mechanism [5–7]. Previously, several miRNAs were reported to exert important roles in DR [8–12]. For example, miR-15a was proved to regulate angiogenesis in DR through targetting VEGF [13]. MiR-451 inhibited retinal pigmental epithelial cells proliferation and migration in proliferative DR [14]. miR-200b and miR-29a were also reported to be involved in the development of DR [15]. These findings indicated significant roles of miRNAs in DR, and we consequently performed a miRNA array in a streptozotocin-induced diabetes model [16], in which several miRNAs were identified up-regulated or down-regulated.

Amongst all down-regulated miRNAs, miR-144-3p was proved to be related to cell proliferation in cancer cells. Li and Jin et al. reported that miR-144-3p induces cell cycle arrest in pancreatic and leukemia cancer cells [17,18]. It was also proved that miR-144 inhibited cell proliferation in breast cancer and melanoma cancer cells [19]. miR-144 was also demonstrated to regulate cell proliferation in hepatocellular carcinoma [20]. However, the role of miR-144 in microvascular epithelial cell proliferation in DR model remains to be illustrated. In the present study, we found that miR-144-3p significantly inhibited cell proliferation induced by high glucose (HG) through suppressing fibroblast growth factor (FGF)16, suggesting miR-144-3p as a potential target for DR therapy.

## Methods

### Cells and treatment

Human umbilical vein endothelial cells (HUVEC) and human retinal endothelial cells (HREC) were purchased from American Type Culture Collection (Manassas, VA, U.S.A.) and maintained in Dulbecco’s Modified Eagle’s medium medium, which were supplemented with 10% fetal bovine serum and 1% penicillin-streptomycin solution (Hyclone, UT, U.S.A.). HREC cells were maintained as the methods described in our previous study. Cells were maintained in a 5% CO_2_ humidified incubator at 37°C. After transfection with miR-144-3p mimics or inhibitors, cells were exposed to different doses of glucose treatments and subjected to next experiments.

### MiRNA oligonucleotides and inhibitors transfection

We transfected miR-144-3p mimics or inhibitors (Genemeditech, Shanghai, China) to increase or inhibit miR-144-3p as described in our previous study. Briefly, HUVEC or HREC cells were seeded in six-well plates at the concentration of 2 × 10^5^. According to the manufacture’s instructions, 50 nM mimics oligonucleotides or 100 nM inhibitors were transfected into cells by using Lipofectamine 3000 transfection reagent (Invitrogen, U.S.A.). At 48 h after transfection, cells were collected for quantitation or treated with glucose.

### RNA extraction and RT-PCR

RNA extraction and real-time PCR (RT-PCR) was conducted as previously described [21]. Briefly, total RNA was extracted by using a TRIzol reagent (Invitrogen) according to the manufacture’s instructions. After then, 2 μg of total RNA was used for reverse transcription to cDNA by using miRNA cDNA synthesis kit (Takara, Dalin, China). RT-PCR for both miRNA and mRNA was performed in ABI 7300 RT-PCR system. U6 and β-actin were used as internal control for miRNA and mRNA expression, respectively. Primers used for VEGF, F:gagttaaacgaacgtacttgcaga, R: tctagttcccgaaaccctga.

### Cell count kit-8 assay and cell cycle analysis

A cell count kit (CCK-8) were used to measure cell proliferation after different treatments. Briefly, cells with/without miR-144-3p mimics or inhibitor transfection were seeded in 96-well plates and then exposed to HG treatment. At 24, 48, and 72 h post-treatment, cells were incubated with 10 μl CCK-8 solution for 3 h in a CO_2_ incubator. Absorbance was measured at 570 nm using a microplate reader (Bio-TEK, Winooski, VT, U.S.A.). For cell cycle analysis, cells were fixed with ethanol at 12 h after high glucose treatment. After RNAase treatment, cells were stained with PI and analyzed in a flow cytometry (BD, U.S.A.) as previously described [22].

### Western blot assay

Western blot were used to detect protein expression and phosphorylation as described previously [21]. In the present study, the following primary antibodies were used: VEGF (Cell signaling Tech., US; 1:1000), p-Akt (Cell signaling Tech., US; 1:1000), p-p38 (Cell signaling Tech., US; 1:1000), p-JNK (Cell signaling Tech., US; 1:1000), GAPDH (Cell signaling Tech., US; 1:2000). Protein bands were detected using the SuperSignal West Pico Chemiluminescent Substrate (Thermo Fisher). The density of each band was analyzed by ImageJ software.

### Elisa assay

Secretary VEGF and FGF16 concentration were determined by using an ELISA kit (Westang Tech, Shanghai, China), according to the manufacturer’s instructions. Absorbance was measured at 570 nm using a microplate reader.

### Wound-healing assay

Wound-healing assay was used to measure cell migration capacity in response to HG treatment. Briefly, after transfection, cells were plated in six-well plates, and 24 h later, cells were scratched gently and slowly with a new 1 ml pipette tip across the center of the well. At 48 h later, images were taken with a microscope (Nikon, Japan). And the distance between two lines was measured and quantitated.

### Target prediction and luciferase reporter assay

A Targetscan 7.0 tool was used for prediction of miRNA target. After that, 3′UTR of FGF16 or a mutated sequence within the predicted seed sequence were cloned into a PGL3 promoter vector in a method previously reported [23]. Finally, firefly and Renilla luciferase activities were measured using a dual-luciferase assay kit (Promega) as previously described [21].

### Statistical analysis

Data were expressed as mean ± S.E.M. The differences between groups were analyzed by using one-way ANOVA followed by Dunnett’s multiple comparisons. The *P*-values <0.05 were considered statistically significant. All experiments were conducted for three independent times.

## Results

### HG treatment down-regulated miR-144-3p level

**Figure 1:**
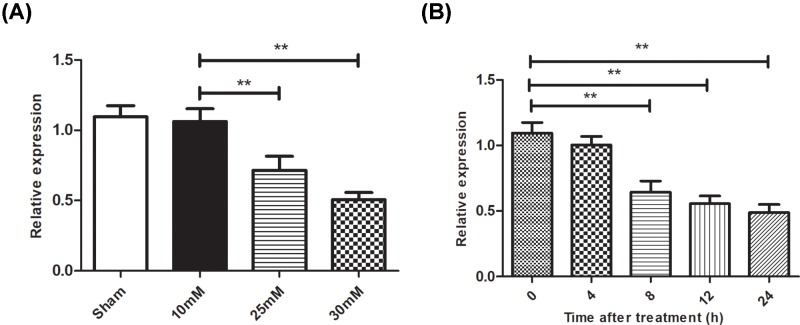
HG treatment reduced miR-144-3p level in HUVEC cells **(A)**: HUVEC cells were exposed to different concentrations of HG treatment for 12 h, after which expression of miR-144-3p were measured by RT-PCR. **(B)**: expression of miR-144-3p were measured at different time post HG treatment. **P<0.01 versus the control groups.

In previous study, our miRNA array data revealed that miR-144-3p was down-regulated in DR model [16]. Subsequently, we used a RT-PCR assay and confirmed that miR-144-3p reduced gradually during the induction of DR model. In the present study, we found that HG treatment reduced miR-144-3p level in a concentration dependent manner (Figure 1A). At 8 h post HG treatment, miR-144-3p decreased to 63% compared with control group (Figure 1B).

### MiR-144-3p inhibited high glucose-induced cell proliferation

**Figure 2:**
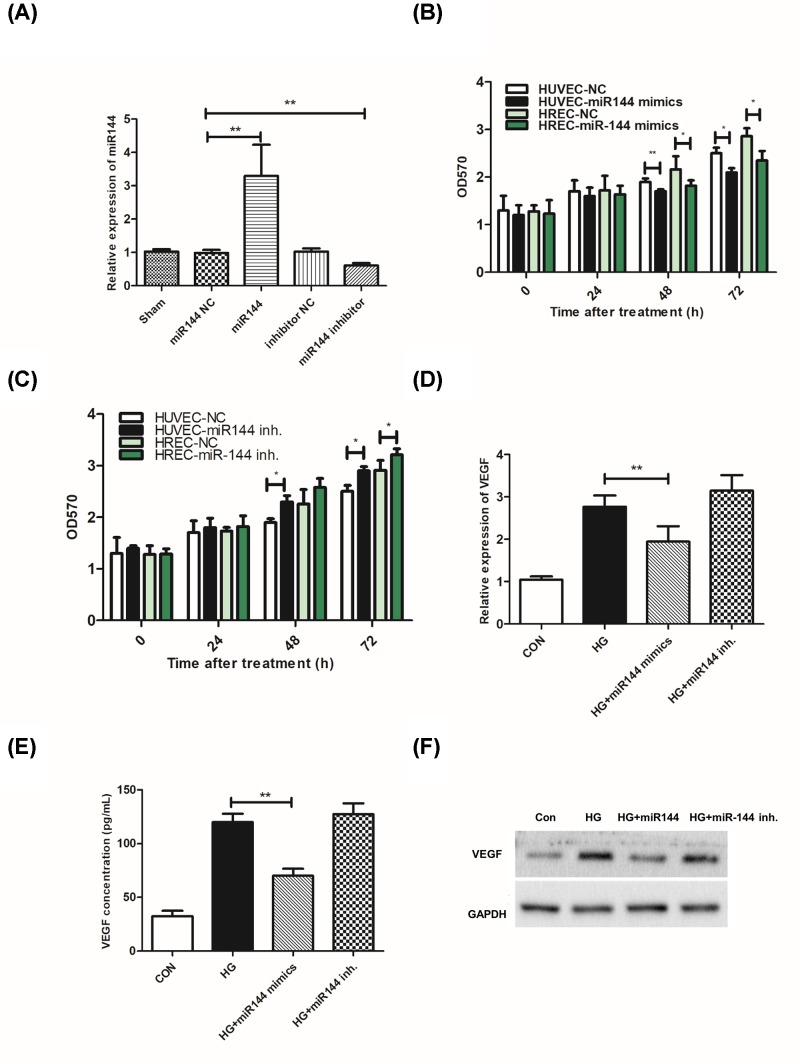
miR-144-3p inhibited cell proliferation in response to HG treatment **(A)**: expression of miR-144-3p in cells transfected with miR-144 mimics or inhibitor. **(B,C)**: cell proliferation was detected by CCK-8 assay in cells transfected with miR-144 mimics or inhibitor. **(D–F)**: miR-144-3p affected secretory VEGF level, mRNA level, and protein level in response to HG treatment. *P<0.05, **P<0.01 versus the control groups.

MiR-144-3p mimics or inhibitor were used to up-regulate or inhibit the level of this miRNA (Figure 2A). In both HUVEC and HREC cells, we found that miR-144-3p mimics significantly inhibited HG induced proliferation, while its inhibitor promoted cell proliferation (Figure 2B,C). Next, we examined the effects of miR-144-3p on VEGF level after HG treatment. Our data showed that the up-regulation of VEGF mRNA level and secretory protein were reduced in miR-144-3p mimics treated groups (Figure 2D–F).

### miR-144-3p reduced cells in S phase and suppressed cell migration

**Figure 3:**
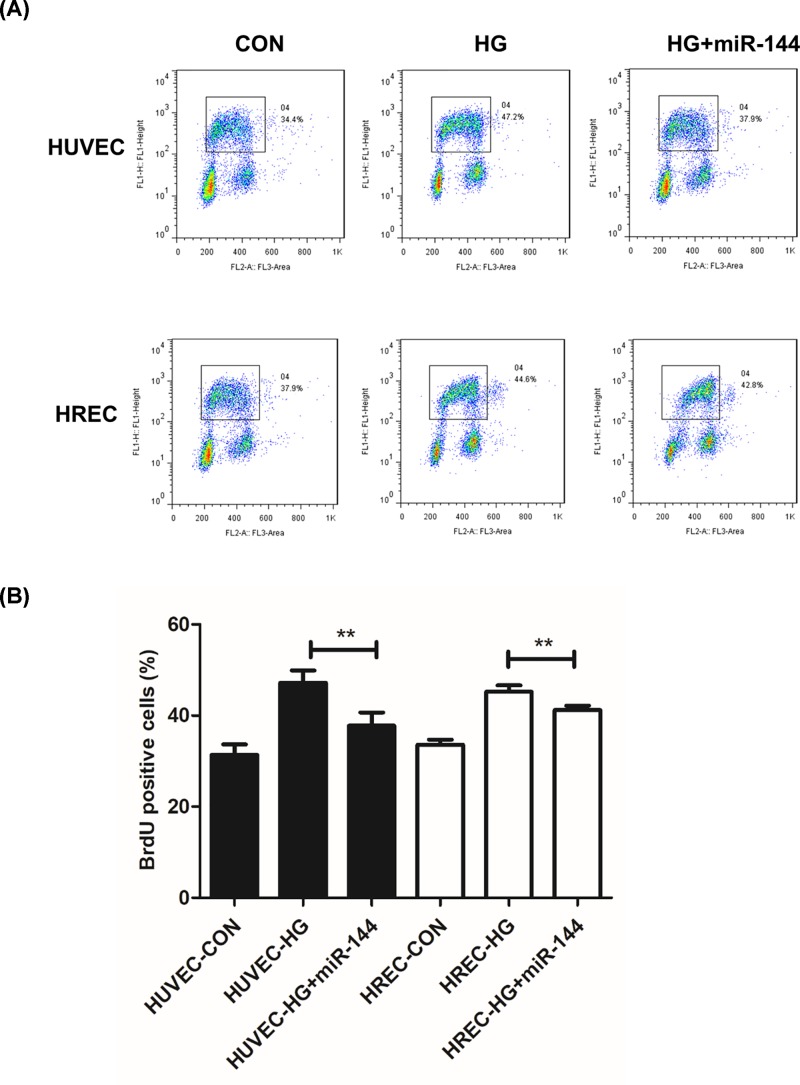
miR-144-3p reduced BrdU positive cells and attenuates cell cycle progression **(A)**: representative images of flow data of BrdU labeled cell cycle in different treated groups. **(B)**: columns of BrdU positive cells were quantitated in different groups. **P<0.01 versus the HG groups.

**Figure 4:**
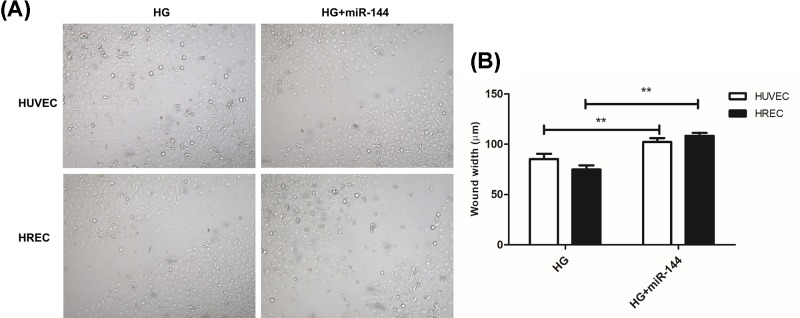
miR-144-3p suppressed HG-induced cell migration **(A)**: representative images of cells at 48 h after scratch in HUVEC and HREC cells. **(B)**: quantitative analysis of the distance between two lines in cells after scratch. **P<0.01 versus the HG groups.

S phase percentage represents the proliferating cell population. We used a 5-Bromo-2-deoxyUridine (BrdU) labeled cell cycle assay to determine the cells in S phase. It was found that HG induced an increase in proliferating cells, which was significantly suppressed by miR-144-3p mimics. However, miR-144-3p inhibitor showed little influence on cell cycle progression when used together with HG. (Figure 3A,B). In wound-healing assay, we found that miR-144-3p significantly inhibited cell migration induced by HG treatment in both HUVEC and HREC cells (Figure 4A,B).

### MiR-144-3p directly targetted FGF16 through a 3′UTR dependent manner

**Figure 5:**
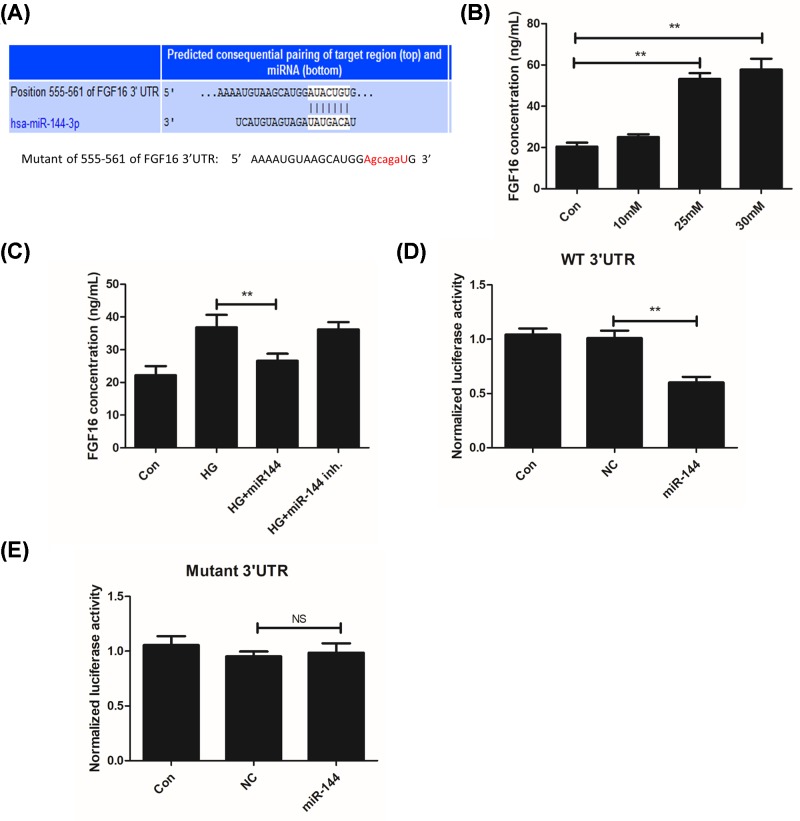
miR-144-3p directly target FGF16 gene through a 3′UTR dependent manner **(A)**: Targetscan prediction of FGF16 as miR-144-3p target and seed sequence mutation procedure. **(B)**: FGF16 level in cells after treatments with different concentration of glucose. **(C)**: FGF16 level in cells with miR-144-3p and/or HG treatment groups. **(D,E)**: miR144-3p represses the luciferase activity of wt FGF16 3′UTR but not on mutant group. **P<0.01 versus the HG groups.

Through Targetscan 7.0 tool, a binding site of miR-144-3p was found in the sequence of FGF16 3′UTR (Figure 5A). Then we determined the changes of FGF16 in response HG treatment, and found that HG induced FGF16 level in a concentration dependent manner (Figure 5B). Moreover, we found that HG induced FGF16 was inhibited by miR-144-3p mimics (Figure 5C). As miRNA regulates its target gene through a 3′UTR dependent mechanism, we constructed a wild-type expression vector and a mutated sequence in a pGL3 luciferase reporter vector. It was found that miR144-3p significantly reduced the luciferase activity in the wild-type sequence, but not in the mutant group (Figure 5D,E).

### FGF16 and MAPK signaling pathway was critical for HG-induced cell proliferation

**Figure 6:**
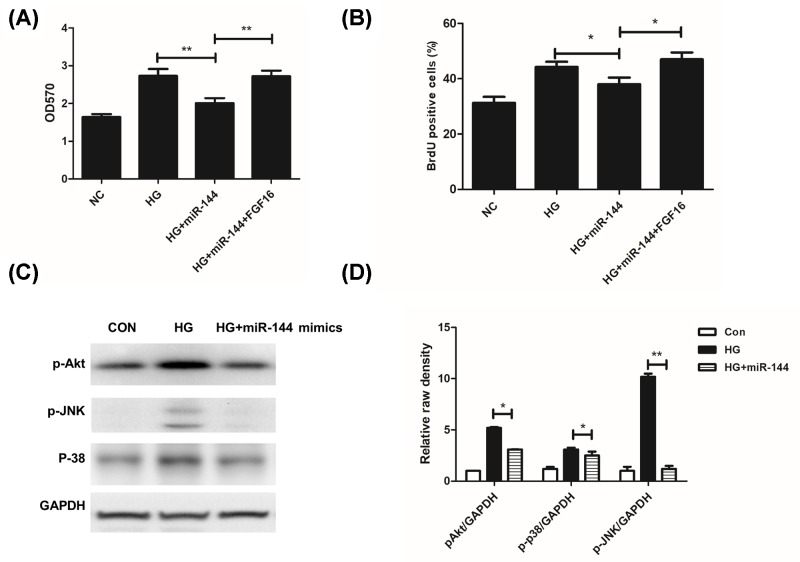
FGF16 and MAPK signaling pathway was critical for HG-induced cell proliferation **(A)**: FGF16 reversed cell proliferation inhibition caused by miR-144-3p. **(B)**: FGF16 treatment increased BrdU positive cells in miR-144 treated group. **(C,D)**: miR-144-3p inhibited MAPK activation by HG. *P<0.05, **P<0.01 versus the control groups.

**Figure 7:**
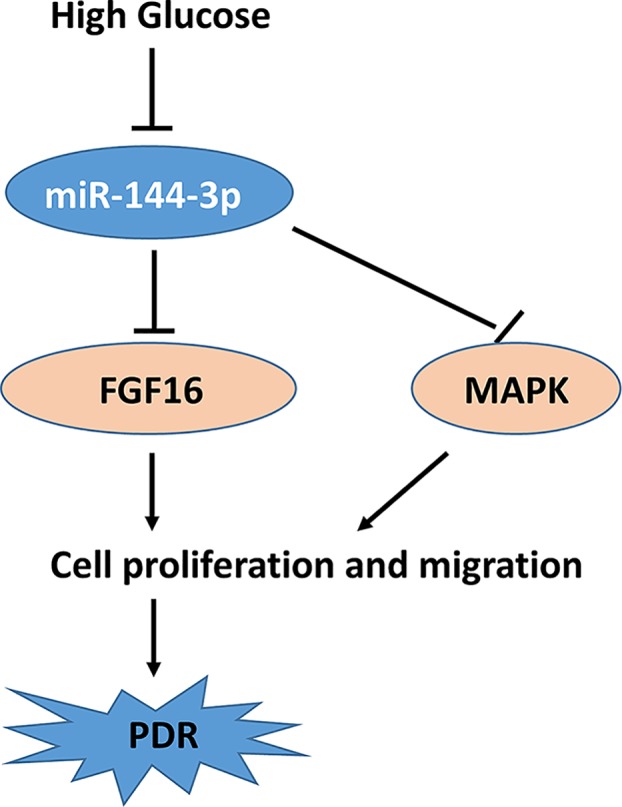
Schematic diagram of the role of miR-144-3p and FGF16 in HG induced cell proliferation and PDR

We demonstrated that FGF16 rescued the inhibition of miR-144-3p on cell proliferation (Figure 6A,B). Finally, we detected the changes of MAPK signaling pathway, which is closely related to cell proliferation. We found that HG significantly triggered the phosphorylation of p38, JNK, and Akt. However, miR-144-3p significantly attenuated the activation of MAPK (Figure 6C,D). These findings indicated that HG induced down-regulation of miR-144-3p, which suppressed cell proliferation through inhibiting FGF16 and MAPK signaling pathway (Figure 7).

## Discussion

DR is one of the most common complications of diabetes mellitus (DM), which often leads to blindness [1,2]. In our previous study, we have performed a miRNA microarray analysis in a rat DM model, in which several miRNAs were found up- or down-regulated [16]. Amongst all, miR-144-3p was gradually down-regulated during the development of DM. Besides, miR-144-3p was also reported to be involved in cell proliferation of many cancer cells [24,25]. In the present study, we confirmed that HG reduced the expression level of miR-144-3p, which is consistent with our microarray data. Moreover, miR144-3p inhibited cell proliferation, cell migration, and cell cycle progression. Finally, we predicted and proved that miR-144-3p directly targetted FGF16 in a 3′UTR dependent manner.

According to pathological progression, DR could be divided into non-proliferative and proliferative subtypes [2]. HG induced endothelial cell proliferation was considered as a key process in the development of proliferative DR [1,2]. In our previous study, we have found that miR-144-3p was down-regulated in rat DR model [16], which was further confirmed in the present study. Surprisingly, miR-144-3p has been shown to exert proliferation inhibitory effects in several cancer cells. For example, miR-144 was found down-regulated in bladder cancer and suppressed cancer proliferation [24]. MiR-144 was also proved to suppress the growth of esophageal squamous cell carcinoma through targetting COX2 [26]. MiR-144 inhibited proliferation of multiple myeloma via suppressing c-MET [25]. In our present study, we found that miR-144-3p mimics significantly inhibited HG induced proliferation of both HUVEC and HREC cells. We performed cell cycle analysis by BrdU labeling, and found a significant decrease in S phase cells. These data showed that HG induced cell proliferation, which is significantly attenuated by miR-144-3p. Our findings indicated that down-regulation of miR-144-3p in response to HG might account for the proliferation promoting effect.

Mechanistically, we predicted a potential target of miR-144-3p, FGF16 through a Targetscan tool. FGFs are multifunctional proteins with a wide variety of effects, including regulatory, morphological, and endocrine effects [27–29]. Amongst all, FGF1 and FGF2 are critical for endothelial cell proliferation and are even moDre potent angiogenic factors than VEGF or PDGF [27,28]. FGF16 is a novel characterized factor of FGF family, and has also been proved to be required for cell proliferation [30,31]. In our cell model, we found that HG induced the expression of FGF16, which was significantly inhibited by miR-144. The luciferase assay also demonstrated that miR-144-3p directly binds to the 3′UTR of FGF16, indicating a role of miR-144 in regulating FGF16. FGF16 treatment also rescued the inhibitory effects of miR-144 on cell proliferation. It was demonstrated that FGF16 regulate MAPK pathway in cancer cells, which is also critical for cell proliferation [32]. We found that miR-144-3p reduced HG-induced activation of MAPK.

The exact mechanism underlying HG induced down-regulation of miR-144 is unclear. We are also working to explore the mechanism of HG induced down-regulation of miR-144. Our ongoing work focussed on two aspects: (1) Transcriptional factors involving miR-144. We focussed on paired box gene 4 (PAX4), which was reported to be located on chromosome 7q32 and exert a function as transcriptional modulator on miR-144 [33]. Besides, PAX4 also regulates insulin activity as well as β cell function and cell proliferation [34]. (2) lncRNA was proved to be competing endogenous RNA to spouse miRNA, which decrease miRNA levels. For example, it was reported that lncRNA TUG spoused with miR-144 and promoted cell proliferation and migration [35]. On the other hand, down-regulation of miR-144 upon hyperglycaemia provides novel clues in the following aspects: (1) down-regulation of miR-144 might provide a prognostic molecular marker for the progression of DR; (2) our finding provides a novel possible therapeutic strategy for DR through exogenous delivery of miR-144; and (3) miR-144-FGF16 axis provides a novel mechanism for hyperglycaemia induced DR.

In conclusion, we demonstrated that miR-144-3p was down-regulated in DR model and HG treated cells. MiR-144-3p inhibited HG-induced cell proliferation through repressing FGF16. MiR-144-3p and FGF16 signaling pathway also contributed to cell proliferation in a post-translational regulatory mechanism. Our finding provides novel insight into the development of DR, suggesting miR-144-3p as novel targets for DR prevention.

## Abbreviations

BrdU: 5-Bromo-2-deoxyuridine
CCK-8: cell count kit 8
DM: diabetes mellitus
DR: diabetic retinopathy
FGF: fibroblast growth factor
HG: high glucose
HREC: human retinal endothelial cells
HUVEC: human umbilical vein endothelial cells
PAX4: paired box gene 4
REC: retinal microvascular endothelial cell
