# Expression of Concern: MicroRNA-144-3p inhibits High Glucose induced Cell proliferation through Suppressing FGF16

**DOI:** 10.1042/BSR-20181788_EOC

**Published:** 2020-07-02

**Keywords:** cell proliferation, diabetic retinopathy, FGF16, miR-144-3p

The Editorial Office has been made aware of potential issues surrounding the scientific validity of this paper, hence has issued an expression of concern to notify readers whilst the Editorial Office investigates.

